# The effects of virtual reality environmental enrichments on craving to food in healthy volunteers

**DOI:** 10.1007/s00213-023-06462-z

**Published:** 2023-09-11

**Authors:** Giulia Benvegnù, Alessandro Piva, Camilla Cadorin, Vanessa Mannari, Matteo Girondini, Angela Federico, Stefano Tamburin, Cristiano Chiamulera

**Affiliations:** 1 Department of Diagnostics and Public Health, University of Verona, Verona, Italy; 2 Department of Neurosciences, Biomedicine, and Movement, University of Verona, Verona, Italy

**Keywords:** Cue-reactivity, Craving, Environmental enrichment, Healthy volunteers, Palatable food, Virtual reality

## Abstract

**Rationale:**

Environmental enrichment (EE) is a non-pharmacological approach that has been shown to be effective in reducing food-taking in rats. Studies in human volunteers are still in their infancy, given the difficulty to translate the complexity of EE in clinical practice. Virtual reality (VR) is a promising methodological approach, but no study has yet applied it to model and test EE in humans.

**Objectives:**

The present study is the first to assess the effects of virtual EE on craving for palatable food.

**Methods:**

Eighty-one healthy volunteers (43 women) were divided into three groups: (i) exposure to a virtual EE (VR-EE), (ii) exposure to a virtual neutral environment (VR-NoEE), and (iii) without exposure to VR (No VR). Craving for palatable food at basal level and evoked by neutral and palatable food images was assessed before and after the VR simulation. Behavior during VR exposure and subjective measures related to the experience were also collected.

**Results:**

VR-EE group showed a significantly greater decrease in pre-post craving difference compared to No VR for all assessments and at basal level compared to VR-NoEE. Interestingly, an inverse correlation between craving and deambulation in the VR simulation emerged in VR-EE group only.

**Conclusions:**

The study highlighted the feasibility of exposing human subjects to an EE as a virtual simulation. Virtual EE induced effects on basal craving for food that suggest the potential for further improvements of the protocol to extend its efficacy to palatable food cues.

**Supplementary information:**

The online version contains supplementary material available at 10.1007/s00213-023-06462-z.

## Introduction

Our society is filled with an abundance of highly palatable, food of low nutrition value that carry a high risk for initiation, maintenance and seeking of the intake of this “junk food” (Ramirez 1990). The rewarding and hedonic properties of highly palatable “unhealthy food” may contribute to increase the incentive salience of associated stimuli and spaces, thus increasing the risk of cue reactivity and craving through Pavlovian, classical conditioning (Sun and Kober 2020). Food reward shares common neurobiological and neurophysiological mechanisms with drug reward (Asmaro and Liotti 2014; Morales and Berridge 2020). Cue-reactivity to food is similar to drug cue-reactivity, and it is a phenomenon characterized by an array of physiological, psychological, and behavioral responses (Sobik et al. 2005), such as changes of galvanic skin conductance, heart rate, craving, food seeking, and binge eating, and its occurrence predicts weight gain (Boswell and Kober 2016). Based on this analogy to drug cue-reactivity and drug-seeking, palatable food cue-reactivity is investigated by using similar experimental paradigms and models (Gearhardt et al. 2011; Alonso-Alonso et al. 2015). Several studies by using food cues presentation in laboratory setting (e.g., images or video) have characterized the brain mechanisms (Tang et al. 2012) and processes underlying food cue-reactivity and the correlates in terms of emotional responses, weight, fasting status, and other conditions (see for a meta-analysis Boswell and Kober 2016). Interestingly, some studies have shown that brain correlates of food craving differ in patterns of activation and inhibitory control of hunger between gender, suggesting better cognitive control of self-reported desire to eat in men compared to women (Wang et al. 2009).

Different therapeutic approaches for addictive behaviors (i.e., pharmacological, behavioral, psychosocial) have been shown to own significant efficacy, with further improvement when combined into a multifactorial intervention (De Crescenzo et al. 2018; Volkow and Boyle 2018). Among non-pharmacological approaches, preclinical studies suggested the efficacy of environmental enrichment (EE) to reduce drug or food-taking and -seeking (Grimm et al. 2008, 2016; Solinas et al. 2010, 2021), thus representing a potential coadjuvant strategy for addiction (Galaj et al. 2020). These effects of EE have been associated to changes in neurotrasmitters, neurotrophins, and transcription factors in brain areas known to be involved in reinforced behaviors (for a recent review see Solinas et al. 2021).

EE is defined as “a combination of complex inanimate and social stimulation’’ (Rosenzweig et al. 1978) and consists in a spatial contextual environment that stimulate motor, sensory, and cognitive processes. Animal models of EE are characterized by a complex housing cage with a variety of objects, such as running wheels, tunnels, and novel toys, which stimulate the animals by providing the opportunity to perform a wide species-specific behavioral repertoire (Nithianantharajah and Hannan 2006). Several animal studies showed that EE was able to induce changes in brain structure and functions, including behavioral changes ranging from learning and memory enhancement to decrease of anxiety and depressive-like symptoms (Rosenzweig and Bennett 1996; van Praag et al. 2000; Laviola et al. 2008).

The main critical issues are how the potential efficacy of EE translates to clinical practice, and how the multidimensional enrichment can be applied in a practical and feasible manner, given the species-specificity in animals and probably the subject-specificity in humans. Although the therapeutic potential of EE has been investigated for different neuropsychiatric disorders (for a review Pang et al. 2019; Galaj et al. 2020; for a meta-analysis Wang et al. 2014), it is however difficult to set up and reproduce EE protocols of inherent complexity in humans. Sensorimotor and cognitive stimulation—similar to animal models described above—with computer games and/or task are used in stroke rehabilitation (e.g., Janssen et al. 2012; Anåker et al. 2017; McDonald et al. 2018). Some studies have shown that video-game exercises improve intervention programs for opioid dependence (Cutter et al. 2014; Abroms et al. 2015) or affect brain correlates of addictive behaviors (Cole et al. 2012; Skorka-Brown et al. 2014, 2015), but these computer-aided interventions do not mimic the complexity of EE conditions. Virtual reality (VR), a technology that creates a state of immersion closer to the real situation and allows for controlled assessment of neuropsychological and behavioral responses, is a promising methodological approach in addictive behaviors (Hone-Blanchet et al. 2014). VR has been widely used for investigations on craving for food (Ledoux et al. 2013; Ferrer-Garcia et al. 2015, 2017; Pla-Sanjuanelo et al. 2015), but none of VR studies on food or drug cue-reactivity incorporated EE in the simulation. Furthermore, it is important to remind that the effects of EE on food craving in healthy volunteers or on drug craving in drug addicts may be mediated by different mechanisms in spite of the common clinical outcome, i.e., craving level.

The main aim of this study is to assess the effects of virtual EE sensorimotor and cognitive stimulation on craving for palatable food induced by computer images compared to neutral images in healthy volunteers. Control groups included VR immersion in a non-EE simulation and no-VR. The secondary aim was to explore the correlation of craving to behavioral and subjective (sense of presence, cybersickness) measures related to the VR experience and to individual traits (food craving, impulsivity, mood, novelty-seeking) measures. We aimed to test the feasibility of an EE condition in VR and to predict VR EE ability to affect food craving, for the first time in a human laboratory setting.

## Methods and materials

### Participants

The experimental sample was composed of healthy volunteers (age 18–65 years), divided into three experimental groups through a semi-randomized distribution. Sample size (27 participants for each group) was estimated by G*Power 3.1 software (36) with an a-priori analysis (α level: 0.05, power level, 1-β: 0.90, small effect size, d: 0.2).

Baseline demographic (age; gender; education; body mass index [BMI]) and personality (food craving, impulsivity, temperament, and character) measures were collected:

- *Food Craving Questionnaire-Trait (FCQ-T).* FCQ-T is a self-administered questionnaire composed of 39 items with a 6-point Likert scale based on a 9-factor model (Cepeda-Benito et al. 2003).
- *Barratt Impulsiveness Scale-11 (BIS-11).* BIS-11 is composed of 30 items with a 4-point Likert scale and measures the level of impulsivity in three areas: attentional impulsivity (IA), motor impulsivity (IM), and non-planning impulsivity (INP) (Fossati et al. 2001).
- *Temperament and Character Inventory (TCI).* TCI is composed of 40 dichotomous items (T/F) belonging to four dimensions: exploratory excitability **(**NS1), impulsiveness (NS2), extravagance (NS3), and disorderliness (NS4) (Fossati et al. 2007).

### Instruments and software

#### Virtual reality tools

HTC-Vive is composed by a viewer (HDM), controllers, and two column movement laser sensors. The HDM has an OLED screen with 2160 × 1200 resolution, 90 Hz frequency, a display angle with 110 degrees that enables to present a 1080 × 1200-pixel image to each eye, and 6 degrees of freedom to track head movements. Body movements are tracked through sensors within the HDM (accelerometer, gyroscope, video camera) and two external laser detectors in an area about 4.5 × 4.5 m. All VR scenarios were created through Blender software and implemented in unity in collaboration with Hybrid Reality s.r.l. (Padua, Italy).

**Fig. 1:**
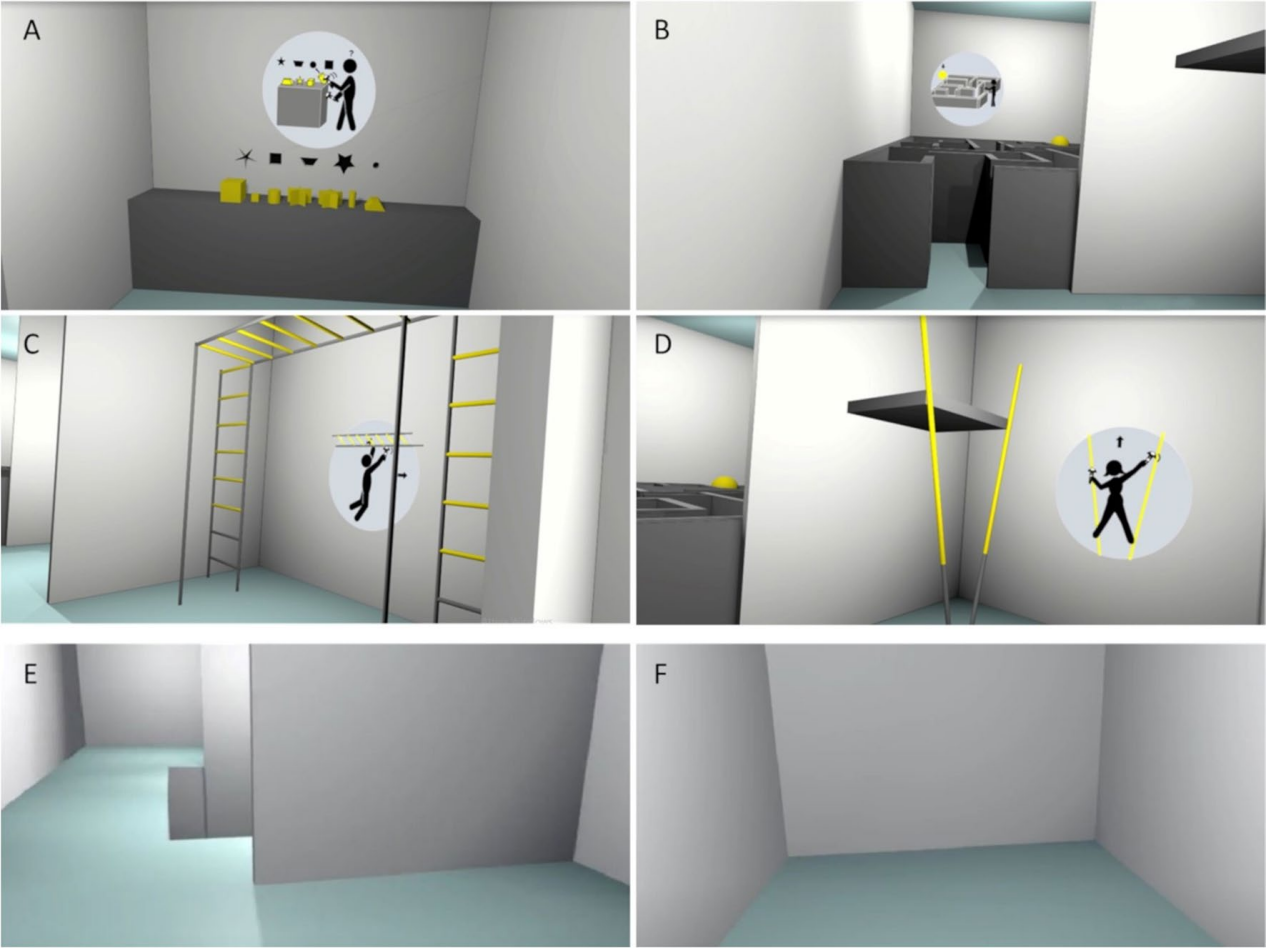
The two virtual environments: the VR-EE scenario, which includes a shape matching task (**a**), a maze exploration task (**b**), a horizontal ladder climbing task (**c**), and a pole climbing task (**d**), and the VR-NoEE scenario (**e**, **f**)

We developed two VR scenarios, namely virtual reality enriched environment (VR-EE) and virtual reality no enriched environment (VR-NoEE). VR-EE was a single open space environment divided into four compartments, which allowed the participant to be engaged in two cognitive and two motor tasks. The cognitive tasks were (a) a shape matching task, including a mental rotation and (b) a visuomotor navigational task in a maze. The motor tasks were (c) interaction with a horizontal ladder and (d) poles (Fig. 1). VR-NoEE was the same an open space with grey walls environment as VR-EE, but all interacting objects were removed, thus not allowing any interaction.

#### E-Prime 2.0.

E-Prime software was used for (1) exposure to neutral objects and palatable food images to control for exposure intervals as well as the presentation order; (2) assessment of craving; and (3) administration of TCI, BIS-11, FCQ-T, and questionnaires to assess secondary outcome measures (see “Methods and materials” section).

Nine neutral object images (neutral) and nine highly palatable and pleasant-tasting food images (cue) were presented to participants. Cue images were pizza slice, sandwich, cheesecake slice, chips, *lasagna*, breaded veal cutlet, *Sacher* cake slice, hamburger, and pancake. All participants were exposed to the same cue images, without considering any individual preference to assess general craving response to a wide range of foods (Ferrer-Garcia et al. 2015). Neutral images were office stationery: calculator, stapler, briefcase, pen, hole-maker, folder, books, clock, and scissors. All images were taken from the FoodCast Research Image Database (FRIDA) (Foroni et al. 2013) and modified with Photoshop to increase their visual impact, in particular, for cue images. Images were presented in random order with intervals of 1000–1400 ms. The time screen presentation for all images was 4000 ms.

#### Behavioral Observation Research Interactive Software (BORIS)

BORIS is a license-free software elaborated by the University of Torino for ethological observation and ethnographical analysis. BORIS was used for visual quantification of the behavior of participants during the exposure to VR environment, using the computer screen output that corresponded to what participants viewed through HMD.

After setting up the test in healthy volunteers (data not included in the present study), we assessed two parameters, namely Interaction and Deambulation. Interaction included the following actions: (a) interacting with yellow-colored objects in the virtual environment by grasping, manipulating, and throwing by pulling the trigger of the hand controller and (b) hit and/or entering the walls. Deambulation included the movements of the participant within the VR space by using hand controller, where each event was teleporting into a participant-pointed white circle on the floor of the virtual room.

Two experimenters independently assessed behavior of participants and pressed a key previously encoded to represent the reference category (i.e., “i” key for Interaction, “d” key for Deambulation) every time the specific behavior was observed in the video recording. Each event was assessed as a single event, and its duration was encoded by pressing the key at its start and end. The measures were the following: (a) number of Interaction or Deambulation events (event), (b) percentage of session time in Interaction or Deambulation (event % time), and (c) average duration of single Interaction or Deambulation events (average duration).

### Primary and secondary outcomes

#### Primary outcome

##### Craving

Craving score was assessed with a numerical rating scale (NRS) formed by a horizontal line, 10 cm long, with intervals numbered from 1 to 10 at regular intervals every 1 cm (Hartrick et al. 2003).

#### Secondary outcomes

##### Presence Questionnaire (PQ)

PQ is based on the presence construct (Witmer and Singer 1998) and is composed of 24 items that assess seven dimensions on a 7-point Likert scale: examination ability, performance self-evaluation, haptic aspects, action quality, interface quality, realism, and sound.

##### Profile of Mood States (POMS)

POMS is composed of 58 items with a 5-point Likert scale that measures six factors: tension/anxiety (T), depression/dejection (D), anger/hostility (A), vigor/activity (V), fatigue/inertia (S), and confusion/bewilderment (C). Total mood disturbance (TMD) score is computed as the sum of the five negative scales (T, D, A, S) minus the positive one (V).

##### Simulator Sickness Questionnaire (SSQ)

SQ assesses sickness intensity experienced during VR exposure and related to nausea, oculomotor, and disorientation through 16 items with a 4-point Likert scale (0–3) (Kennedy et al. 1993).

### Procedures

#### Recruitment

Participants were recruited through online and paper advertisements placed in the main gathering areas of the School of Medicine, Verona University, Italy. The procedures and potential risks associated to the experimental study were clearly and fully explained to the participants, who signed informed consent prior to the experimental sessions. The study was approved by the local academic ethical committee for research in healthy volunteers (*Comitato di Approvazione per la Ricerca sull’Uomo*, CARU, protocol 17/2020) and followed the principles of the Declaration of Helsinki.

Participants were asked to fast during the 2 h preceding the experiment and avoid the intake of psychoactive substances, such as coffee, tea, nicotine, and alcohol.

#### Experimental procedure

**Fig. 2:**
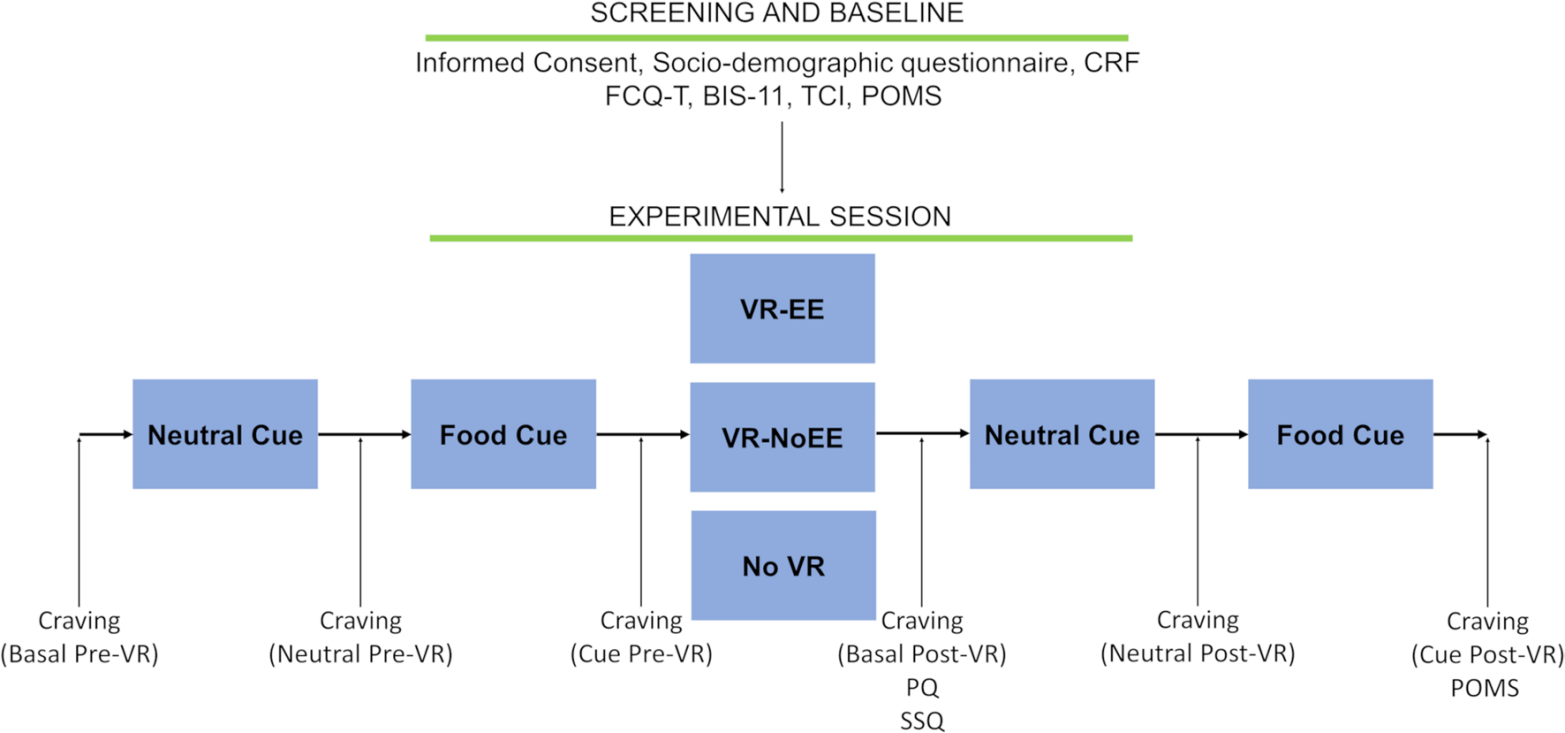
Schematic representation of the procedure. CRF, case report form; FCQ-T, Food Craving Questionnaire–Trait; BIS-11, Barratt Impulsiveness Scale-11; TCI, Temperament and Character Inventory; POMS, Profile of Mood States; PQ, presence questionnaire; SSQ, Simulator Sickness Questionnaire

The experiment lasted about 45 min and consisted of seven phases (Fig. 2):

1. Screening: welcoming the participant, informed consent, COVID-19 statement, privacy form, socio-demographic questionnaire, and case report form (CRF).
2. Baseline assessment: administration of questionnaires (FCQ-T, BIS-11, TCI, POMS).
3. Basal pre-VR assessment: basal craving score assessment.
4. Pre-VR image exposure: presentation of neutral and then cue images (time screen presentation for each image: 4 s), each followed by craving score assessment (neutral pre-VR, cue pre-VR timepoints).
5. VR session: exposure to VR consisting of a 1-min tutorial to familiarize with the device, and then a 4-min VR immersion for VR-NoEE and VR-EE groups. Participants in the No VR group sat at the desk and during the 5-min period were free to do whatever they wanted, except using mobile phones.
6. Post-VR assessment: basal craving score assessment, and PQ and SSQ administration (VR-EE, VR-NoEE groups).
7. Post-VR image exposure: presentation of neutral and then cue images (time screen presentation for each image: 4 s), each followed by craving score assessment (neutral post-VR, cue post-VR timepoints), followed by POMS administration.

### Data analysis

Statistical analysis was carried out with the IBM SPSS version 20.0 and GraphPad Prism version 9.1.0.

For the comparison of baseline and demographic variables, the nonparametric Kruskal–Wallis H test and post-hoc Dunn’s multiple comparisons test were used for continuous variables and the chi-squared test for dichotomous ones.

Three-way repeated-measure ANOVA was applied to craving score with the between-groups variable GROUP (VR-EE, VR-NoEE, No VR) and the within-group variables IMAGE (basal, neutral, cue) and TIME (pre-VR, post-VR). Between-groups post-hoc comparisons were performed with least significant difference (LSD) test on the pre-post differences in craving scores (Δ craving) for each IMAGE condition. Post-hoc Sidak’s test was used to compare differences in craving scores across different IMAGE conditions within each group.

Mann–Whitney *U*-test was used to compare PQ and SSQ scores and the BORIS measures for Deambulation and Interaction between VR-EE and VR-NoEE groups.

Correlation between craving scores and the percentage of session time in Deambulation (event % time) were tested with Pearson’s *r*.

One-way ANOVA was applied to BIS-11, FCQ-T, and TCI scores.

*P* < 0.05 (two-tailed) was taken as the significance threshold for all the tests.

## Results

### Participants

A total of 116 subjects were screened, of which 35 were excluded because of acute disease (*N* = 1), chronic disease (*N* = 11), lack of interest (*N* = 6), incompatibility with working hours (*N* = 7), lack of reward for participating in the study (*N* = 6), current therapy with psychiatric drugs (*N* = 2), pregnancy (*N* = 1), and refusal to give reasons for non-participation (*N* = 1), and 81 were included.

Demographic (age: *H* [2] = 1.147, *p* = 0.563; gender: *χ*^2^ [2] = 3.073, *p* = 0.215; education: *χ*^2^ [4] = 2.065, *p* = 0.723; BMI: *H* [2] = 1.442, *p* = 0.486) and personality (FCQ-T, BIS-11, TCI scores) variables did not differ significantly between groups (Table 1).

**Table 1:** Baseline demographic and personality measures

	No VR (*N* = 27)	VR-EE (*N* = 27)	VR-NoEE (*N* = 27)	Total (*N* = 81)
Age (y)^†^	26.9 ± 6.8; 19–52	27.4 ± 7.7; 19–58	30.0 ± 9.7; 18–56	28.1 ± 8.2; 18–58
Gender (M/W)	14/13	15/12	9/18	38/43
Education (HS/BSc/MSc)	10/6/11	9/3/15	11/5/11	30/14/37
BMI (kg/m^2^)^†^	22.5 ± 3.0; 17.9–29.0	22.0 ± 2.6; 16.1–27.7	21.8 ± 3.4; 16.1–32.7	22.1 ± 3.0; 16.1–32.7
FCQ	95.1 ± 22.1	97.3 ± 21.8	85.8 ± 21.1	92.7 ± 22.0
BIS-11				
Total	57.5 ± 7.8	56.8 ± 10.5	57.4 ± 11.3	57.3 ± 9.9
IA	14.5 ± 4.3	12.6 ± 3.0	15.6 ± 4.1	14.3 ± 4.0
IM	18.7 ± 2.6	20.1 ± 4.0	19.3 ± 4.6	19.4 ± 3.9
INP	24.3 ± 3.6	24.1 ± 5.2	22.5 ± 5.4	23.6 ± 4.8
TCI				
Total	19.4 ± 5.1	18.0 ± 6.6	18.5 ± 4.3	18.6 ± 5.4
NS1	6.9 ± 2.3	6.2 ± 2.4	6.8 ± 2.3	6.6 ± 2.3
NS2	3.2 ± 1.6	3.6 ± 2.8	3.0 ± 1.7	3.3 ± 2.1
NS3	4.2 ± 1.8	4.1 ± 2.0	3.8 ± 2.0	4.0 ± 1.9
NS4	5.2 ± 2.0	4.2 ± 1.8	4.9 ± 1.8	4.8 ± 1.9

^†^mean ± SD; range

*BMI* body mass index, *BSc* bachelor’s degree, *HS* high school, *M* men, *MSc* master’s degree, *No VR* no exposure to VR, *VR-EE* virtual reality enriched environment, *VR-NoEE* virtual reality no enriched environment, *W* women, *FCQ-T* Food Craving Questionnaire–Trait, *BIS-11* Barratt Impulsiveness Scale-11 with, as sub-scales, *IA* attentional impulsivity, *IM* motor impulsivity, *INP* non-planning impulsivity, *TCI* Temperament and Character Inventory with, as sub-scales, *NS1* exploratory excitability, *NS2* impulsiveness, *NS3* extravagance, and *NS4* disorderliness

### Craving score

**Fig. 3:**
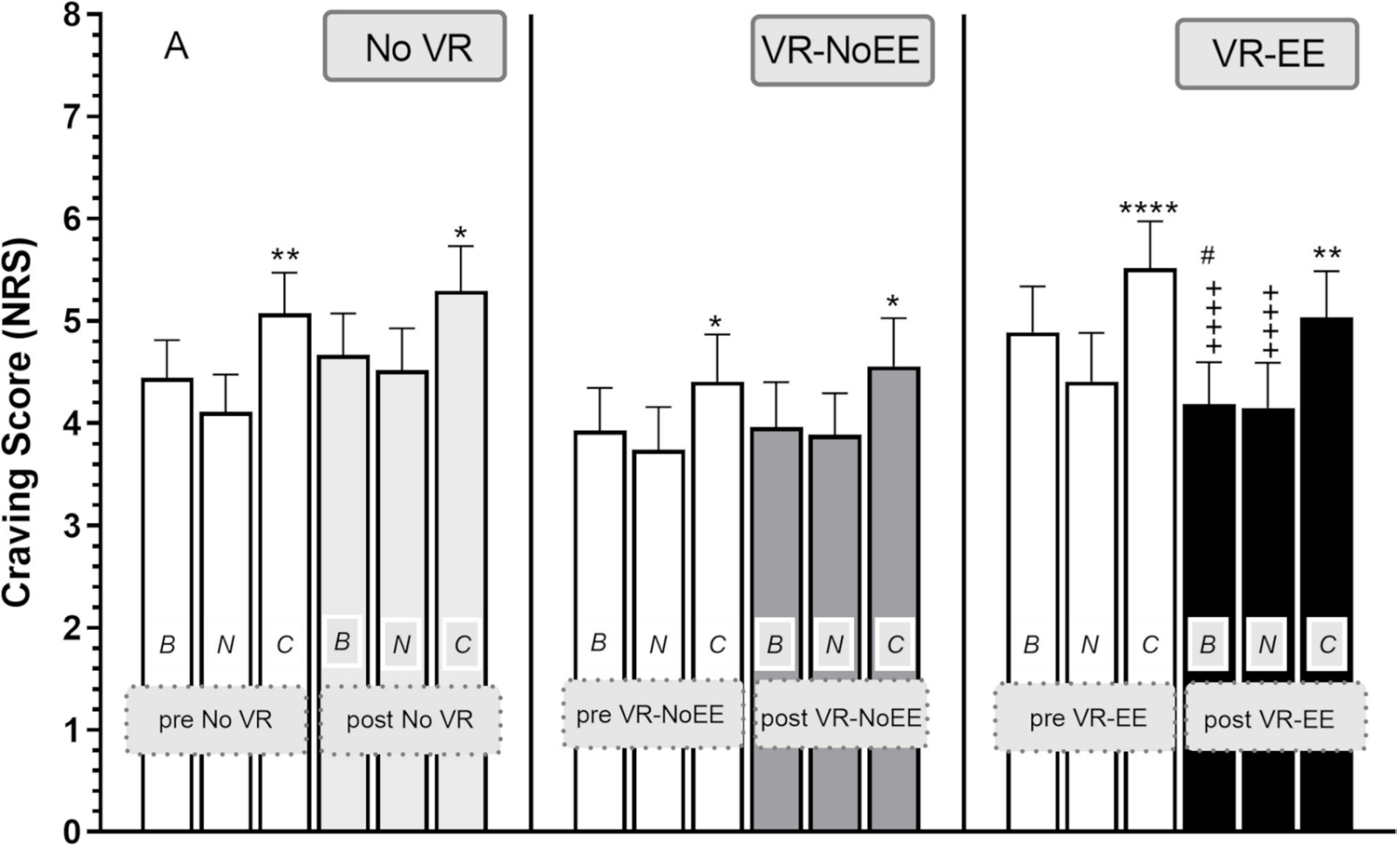
Effects of experimental manipulation on craving scores (means and S.E.M). Craving was assessed at the different assessments timepoints to basal (*B*), neutral (*N*), and cue (*C*) images before (pre-VR: white columns) and after virtual reality administration (post-VR: No VR, light grey columns; VR-NoEE, grey columns; VR-EE, black columns). Horizontal lines equal 1 S.E.M. *(*p* < 0.05), **(*p* < 0.01), and ****(*p* < 0.0001) mark significant post-hoc cue vs. neutral images within-group comparisons (Sidak’s test). #(*p* < 0.05) mark significant post-hoc post-VR vs. pre-VR within-group comparisons (Sidak’s test). +  +  +  +  = *p* < 0.0001 vs. palatable food picture timepoint value cue pre-VR. No VR, no exposure to VR; VR-EE, virtual reality enriched environment; VR-NoE, virtual reality no enriched environment

Figure 3 shows craving scores at different timepoints for the three groups.

Three-way repeated-measure ANOVA showed significant effect of IMAGE (*F* [2, 156] = 56.8; *p* < 0.0001) and significant TIME × GROUP interaction (*F* [2, 156] = 3.7; *p* = 0.029), while the other variables and interaction were not significant.

Post-hoc LSD test showed a significant effect on the pre-post craving difference (Δ craving), in that craving was reduced when comparing VR-EE (basal: − 0.7 ± 1.7; neutral: − 0.3 ± 1.5; cue: − 0.5 ± 1.6) to No VR groups for all IMAGES (basal: 0.2 ± 1.0, *p* = 0.01; neutral: 0.4 ± 0.7,* p* = 0.03; cue:0.2 ± 0.9; *p* = 0.04) and when comparing VR-EE to VR-NoEE for basal craving score (0.04 ± 1.1, *p* = 0.04).

Post-hoc Sidak’s test showed a significant increase of craving score when comparing cue to neutral images in all groups in the pre-VR (No VR: *p* < 0.001; VR-NoEE: *p* = 0.047; VR-EE: *p* < 0.0001) and post-VR (No VR: *p* < 0.01; VR-NoEE: *p* = 0.047; VR-EE: *p* < 0.01) conditions and a significant decrease in basal craving score when comparing post-VR to pre-VR conditions (*p* < 0.028; Sidak’s) in the VR-EE group only.

Given the unbalanced distribution of men and women in one of the groups (VR-NoEE), further analysis was conducted to test for a potential effect of sex on craving. Three-way repeated-measures ANOVAs were performed, separately by group, using SEX (male, female) as the between-group variable and IMAGE (basal, neutral, cue) and TIME (pre-VR, post-VR) as within-group variables.

The main effect of SEX was not significant in VR-EE (*F* [1, 25] = 0.81; *p* = 0.3), VR-NoEE (*F* [1, 25] = 0.002; *p* = 0.9) and No VR (*F* [1, 25] = 0.7; *p* = 0.3). Similarly, the interaction between SEX and TIME or IMAGE was not significant in any of the three groups (Supplemental Information, Table S1).

### VR immersion, activity, sickness, and mood measures

The VR-EE group reported significantly higher PQ overall score compared to the VR-NoEE group (*U* = 122, *p* < 0.0001). When analyzing single PQ dimensions, examination ability (*U* = 191.5, *p* = 0.002), haptic aspects (*U* = 129, *p* < 0.0001), action quality (*U* = 250, *p* = 0.046), realism (*U* = 215.5, *p* = 0.009), and sound (*U* = 128, *p* < 0.0001) were significantly higher in the VR-EE than the VR-NoEE group.

**Fig. 4:**
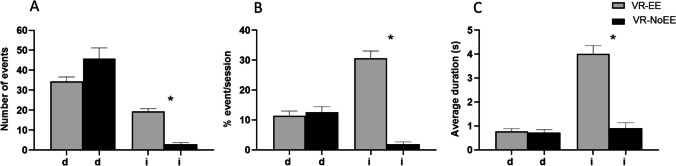
Deambulation (d) and Interaction (i) measures in the VR-EE and VR-NoEE groups (means and S.E.M): number of Deambulation and Interaction events (**a**), percentage of session time in Deambulation and Interaction (**b**), and average duration (in seconds) of single Deambulation and Interaction events (**c**)

Analysis of BORIS measures showed higher Interaction scores in the VR-EE than the VR-NoEE group, (event % time: *U* = 14.5, *p* < 0.0001; duration: *U* = 16, *p* < 0.0001; average duration: *U* = 60, *p* < 0.0001), while Deambulation scores did not differ between groups (Fig. 4).

SSQ did not significantly differ between VR-EE (5.1 ± 5.1) and VR-NoEE (4.1 ± 5.4) groups (Supplemental Information, Table S2).

**Fig. 5:**
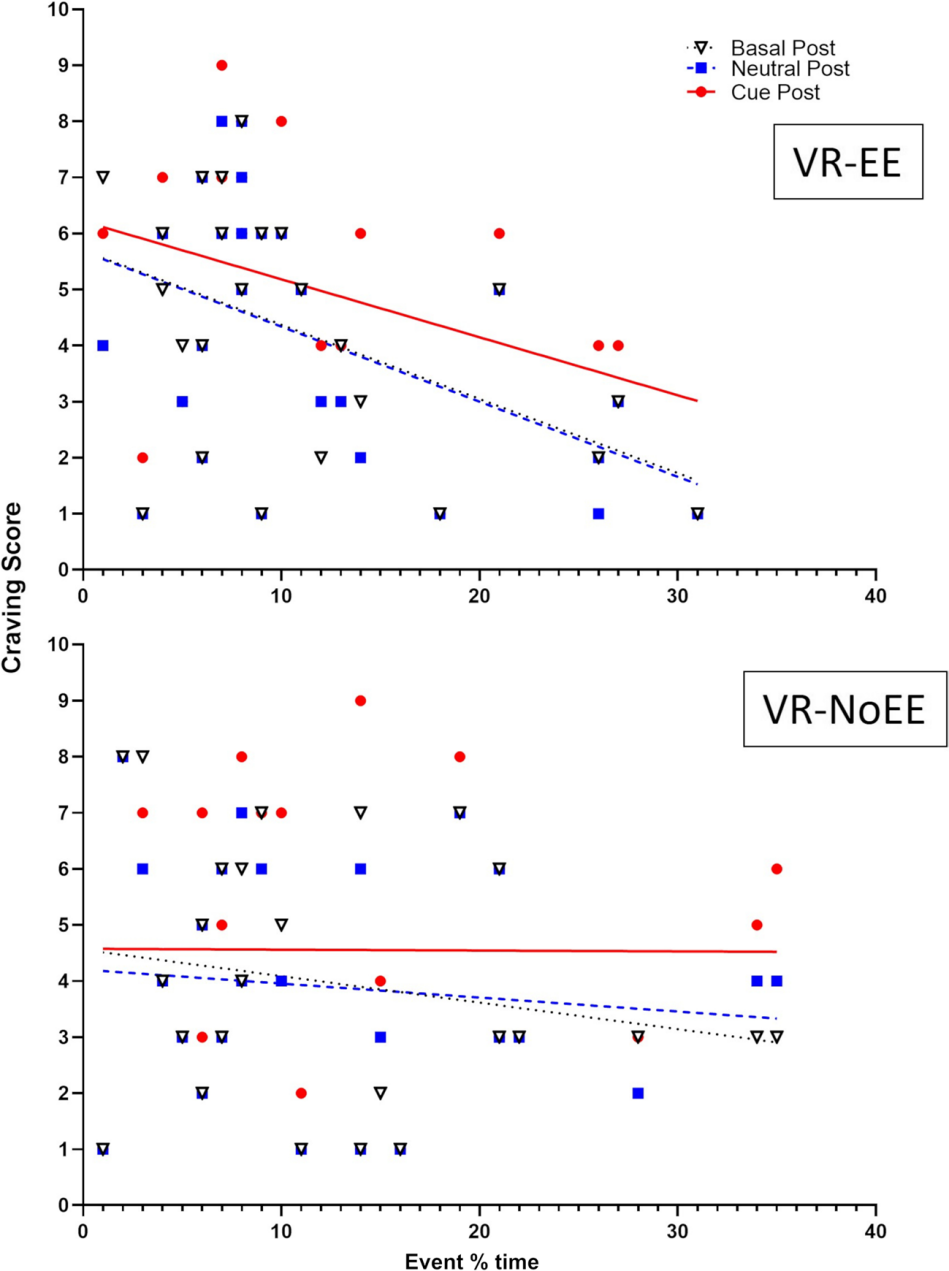
Correlational analysis between activity measures of Deambulation “percentage of session time in deambulation” (event % time; *X*-axis) and craving scores values (*Y*-axis) at post VR assessment timepoints basal (triangle, dotted line), neutral (square, dashed line), and cue (circle, solid line) in the enriched environment (VR-EE; top panel), and standard (VR-NoEE; bottom panel) virtual reality (VR) scenario

However, a correlative pair-wise analysis (Pearson’s *r*) between the measures of Deambulation “event % time” and craving scores at post VR assessment timepoints, showed a significant inverse correlation with craving scores for basal (basal post; *r* =  − 0.50, *p* < 0.01) and neutral (neutral post; *r* =  − 0.4731, *p* = 0.012) and a close to significance trend for food (cue post; *r* =  − 0.36, *p* = 0.064) in the VR-EE but not in the VR-NoEE group (Fig. 5).

The experimental session brought general decrease of POMS with the most significant changes occurring in the VR-EE condition (Table 2). Two-way ANOVA showed significant effect for TIME (*F* [1, 78] = 45.46; *p* < 0.0001) but not for GROUP for POMS questionnaire (TMD score). Regarding POMS subscales, all of them showed a significant effect for factor TIME (Supplemental Information, Table S3). A significant TIME × GROUP interaction emerged for fatigue/inertia (S) (*F* [2, 78] = 3295; *p* = 0.043) and confusion/bewilderment (C) (*F* [2, 78] = 5057; *p* < 0.01) subscales. Sidak’s test compared POMS-bsl to POMS-end and showed significant effect for VR-EE (*S*: *p* < 0.0001; *C*: *p* < 0.0001), VR-NoEE (*C*: *p* < 0.001), and No-VR (*S*: *p* < 0.0001).

**Table 2:** Mood (POMS) measures

	No VR (*N* = 27)	VR-EE (*N* = 27)	VR-NoEE (*N* = 27)	Total (*N* = 81)
POMS-bsl	19.63 ± 25.95**	17.70 ± 35.20***	18.56 ± 35.03**	18.63 ± 31.95
T	6.82 ± 4.92	6.67 ± 5.79	5.59 ± 5.73	6.36 ± 5.45
D	9.67 ± 8.43	6.74 ± 10.08	7.00 ± 8.01	7.80 ± 8.87
A	4.67 ± 5.11	4.67 ± 6.98	4.89 ± 6.93	4.74 ± 6.32
V	16.63 ± 5.68	17.19 ± 4.73	17.70 ± 5.01	17.17 ± 5.11
S	6.56 ± 3.98***	5.74 ± 4.28***	4.93 ± 4.20	5.74 ± 4.16
C	8.56 ± 4.45	11.07 ± 7.94***	11.04 ± 9.18***	10.22 ± 7.46
POMS-end	7.56 ± 21.19	− 0.41 ± 22.60	5.15 ± 23.79	4.10 ± 22.52
T	4.48 ± 4.26	3.93 ± 4.46	3. 70 ± 3.22	4.04 ± 3.98
D	5.44 ± 7.37	2.48 ± 5.85	3.93 ± 5.86	3.95 ± 6.43
A	2.04 ± 3.04	1.00 ± 2.80	3.19 ± 5.02	2.07 ± 3.82
V	16.04 ± 5.59	15.63 ± 5.98	16.48 ± 5.40	16.05 ± 5.60
S	4.00 ± 3.14	2.26 ± 3.65	3.56 ± 4.16	3.27 ± 3.70
C	7.63 ± 4.49	5.56 ± 4.70	5.85 ± 4.69	6.35 ± 4.66

*POMS-bsl* basal Profile of Mood States with, as sub-scales, *T* tension/anxiety, *D* depression/dejection, *A* anger/hostility, *V* vigor/activity, *S* fatigue/inertia, and *C* confusion/bewilderment, *POMS-end* same as above assessed at the end of the experimental session, *VR-EE* virtual reality enriched environment, *VR-NoEE* virtual reality no enriched environment, and *No VR* no exposure to VR.

**(*p* < 0.01) and ***(*p* < 0.001) mark significant post-hoc POMS-bsl vs. POMS-end within-group comparisons (Sidak’s test)

## Discussion

Exposure to EE in a VR simulation reduced basal craving, while standard VR and control groups did not show the same effect. On the other hand, VR-EE was not able to reduce craving evoked by palatable food images. Craving values after exposure to VR-EE but not to the other groups was inversely correlated to Deambulation (event % time) in the virtual environment. These findings suggest that virtually simulated EE simulation reduces basal, not picture-evoked, craving but did not affect the evoked craving response to palatable food.

The FRIDA protocol used in the present study showed reliability to induce craving when subjects were exposed to palatable food images compared to neutral ones. Moreover, EE exposure in the VR simulation, but not NoEE, was able to reduce basal craving and to stimulate virtual activities such as Deambulation and Interaction, with the former inversely correlated to craving scores.

To our knowledge, this is the first example of EE developed and delivered in VR in normal controls. The design of EE in our study was based on animal models, which included sensorimotor, perceptual, and cognitive stimulations (van Praag et al. 2000; Laviola et al. 2008). According to this methodological strategy, we designed a virtual EE that included cognitive stimulation by shape matching and maze orientation tasks, and motor coordination, and stimulated movements with horizontal ladder and poles. We were able to detect that a virtual EE with these components was effective to induce changes in craving for palatable food and to stimulate virtual activities (e.g., exploratory deambulation) differently from the not-enriched virtual immersion in the NoEE group. We could therefore propose that the configurational complexity of our virtual environment and the opportunity of affordances in the VR-EE groups could be considered a valid (virtual) environmental enrichment.

The findings of this study should be discussed in the context of the hypothetical mechanisms underlying the general EE effect and the lack of predicted effect on cue-induced evoked craving. EE has been widely demonstrated to reduced drug and food reward behaviors in animal models (Solinas et al. 2010). EE was shown not only to have a *preventive* effect, that is to reduce or block the acquisition of reinforced behaviors, but also a *curative* action, that is the inhibition of the conditioned response in laboratory animals after the acquisition of reinforced behavior (Solinas et al. 2010). We think that the significant decrease of POMS fatigue scores (dimension that refers to the level of stress and exhaustion) in the VR-EE, but not in the VR-NoEE group, might be due to a “stress-relief” effect. However, considering the short EE exposure applied in our study, we hypothesize that this “stress-relief” effect is due to an acute, presumably transient and short in duration, decrease of arousal levels that hypothetically contribute also to the decrease of basal craving for palatable food.

The significantly higher sense of Presence and Interactions in the VR-EE compared to the VR-NoEE group confirmed the richness of the EE exposure in terms of immersion and interaction. Presence may be defined as a measure of involvement and interactions in the virtual environment, including sensory and attentional processes (Witmer and Singer 1998). Presence level may thus depend on selective attention, degree of sensorial immersion, involvement, and naturalness of action-perception interactions. Interactions are index of participant’s involvement in the VR-EE environment, as expected, compared to the non-enriched VR simulation. Interestingly, although the amount of Deambulation was similar between VR-EE and VR-NoEE, i.e., they had a similar amount of activity, we observed a statistically significant inverse correlation with post-VR basal and neutral craving scores (and a close to significance trend for cue) for the VR-EE but not for the VR-NoEE group. This correlation may be due to a common underlying mechanism or process that is not presumably occurring in the VR-NoEE condition, thus excluding any EE-independent causal relationship between Deambulation and craving. A conservative explanation based on the “stress-relief” hypothesis, is that participants more active in deambulatory movements were less stressed compared to those less active, with a consequent causal effect of stress-relief on craving for food (but not for palatable food). Even if it is not possible to draw any direct causal relationship between Deambulation and craving, the fact that Deambulation occurred temporally before craving assessments may suggest that it could be used as a potential correlational variable with predictive value for craving scores.

There are several limitations in the present study. The aim of the study was not reached, as VR-EE was not able to reduce evoked craving to food; this may be due to different reasons. First, the intensity of evoked craving in our experimental conditions was too strong for being inhibited by VR-EE exposure, i.e., a ceiling cue-reactivity effect. It must be said however that the average values of evoked craving scores were in range of values lower than the maximum. The inclusion in the study of an extra group exposed to a known inhibitory effect on evoked craving for palatable food—for instance a cue exposure therapy manipulation—could have provided a standard effective comparator. Second, the intensity of the enrichment simulation was not sufficient to counteract evoked craving; in that case, the inclusion of a group exposed to longer or richer EE simulation could help assess an EE dose–effect relationship. Finally, it should be also remarked that cue-reactivity to palatable food images was assessed only at the psychological level (i.e., subjective craving score assessment). Neurophysiological measures of cue-reactivity, such as galvanic skin reactions or heart rate variability (Bordnick et al. 2005) may have been more sensitive to change. Remarkably, the different brain activation and inhibition during food stimulation in men compared to women provided the cognitive mechanism that could explain the higher vulnerability to food cues in women (Wang et al., 2019). Further studies should assess whether autonomic measures of cue-reactivity compared to self-reported subjective craving may help to have a further insight into gender differences in inhibiting the desire for palatable food.

The aim of our study was to test the acute, ad hoc, effect of EE on a brief cue reactivity protocol session. Our research question was whether an ad hoc EE “stimulation” might help to reduce situational craving. We were aware of the difference between the modality of EE exposure in our study compared to those investigated in preclinical models of food or drug-seeking and -taking behavior. We applied a short, few minutes exposure to an enriched manipulation that evidently differed from hours to days exposure periods in rodent studies based on models of prolonged drug exposure and/or conditioning training. These differences raise the critical question whether the rationale of our study is the same of preclinical studies or it should be based on different assumptions. Further studies should characterize the mechanisms underlying the acute ad hoc EE effects per se, such as for instance the modulation of affective response (i.e., stress-relief?).

This study has some strength. First, we documented the feasibility to expose human subjects to an EE virtual simulation. Under these conditions, we showed that VR-EE was able to increase sense of presence—a measure of immersion and involvement—compared to VR-NoEE group. Second, the fact that we were able to detect an effect of virtual EE on craving for food suggest that modification of the protocol in terms of VR-EE duration or intensity might result in an efficacy on craving for palatable food cues.

Future studies should explore different features of EE modeled in VR that could affect motivational and emotional responses linked to maladaptive appetitive and addictive behaviors. VR could thus offer not only a technology to model complex naturalistic conditions in the lab, but also offer the opportunity of “digital phenotyping” approaches (Insel 2017) for research (e.g., Rodrigues et al. 2020; Chiamulera et al. 2021) and intervention (Torous and Hsin 2018).

### Supplementary information

Below is the link to the electronic supplementary material.

Supplementary file1 (DOCX 20 KB)
