# Supplementary material for: The effects of virtual reality environmental enrichments on craving to food in healthy volunteers
Source: Psychopharmacology (Berl). 2023 Sep 11;241(1):49–60. doi: 10.1007/s00213-023-06462-z (PMC10774167; doi:10.1007/s00213-023-06462-z)
Supplement: Supplementary file 1 — Supplementary file1 (DOCX 20 KB) [file 213_2023_6462_MOESM1_ESM.docx]

**Article title:** The effects of Virtual Reality Environmental Enrichments on craving to food in healthy volunteers

**Journal**: Psychopharmacology

**Authors:** Giulia Benvegnù, Alessandro Piva, Camilla Cadorin, Vanessa Mannari, Matteo Girondini, Angela Federico, Stefano Tamburin, Cristiano Chiamulera

**Corresponding author:** Giulia Benvegnù, Department of Diagnostics and Public Health, University of Verona, Verona, Italy, [giulia.benvegnu@univr.it](mailto:giulia.benvegnu@univr.it)

**Table S1 Effect of sex on craving (interactions)**

|  | **SEX × IMAGE** | **SEX × TIME** | **SEX × IMAGE × TIME** |
| --- | --- | --- | --- |
| VR-EE  VR-NoEE  No VR | F (2, 50) = 0.74; *p* = 0.4  F (2, 50) = 0.52; *p* = 0.5  F (2, 50) = 0.54; *p* = 0.5 | F (1, 25) = 0.48; *p* = 0.4  F (1, 25) = 0.16; *p* = 0.6  F (1, 25) = 0.99; *p* = 0.3 | F (2, 50) = 0.13; *p* = 0.8  F (2, 50) = 0.62; *p* = 0.5  F (2, 50) = 1.19; *p* = 0.3 |

**Legend.** VR-EE: Virtual Reality Enriched Environment, VR-NoEE: Virtual Reality No Enriched Environment, No VR: no exposure to VR

**Table S2 PQ subscales**

|  | **VR-EE (N = 27)** | | **VR-NoEE (N = 27)** | |
| --- | --- | --- | --- | --- |
|  | **Mean ± SD** | **Median** | **Mean ± SD** | **Median** |
| AdE  AdP  AT  QdA  QdI  R  S | 17.41± 2.46  11.67 ± 1.75  8.55 ± 2.25  21.19 ± 4.15  16.63 ± 2.70  37.85 ± 5.03  12.96 ± 3.64 | 18  12  8  22  17  38  13 | 14.74± 3.31  11.26 ± 1.93  5.22 ± 3.04  18.15 ± 5.47  16.30 ± 2.98  31.70 ± 8.72  7.63 ± 4.33 | 14  12  4  19  16  30  7 |

**Legend.** VR-EE: Virtual Reality Enriched Environment, VR-NoEE: Virtual Reality No Enriched, AdE: Examination Ability, AdP: Performance Self-evaluation, AT: Haptic Aspects, QdA: Action Quality, QdI: Interface Quality, R: Realism and S: Sounds

**Table S3 POMS subscales**

|  | **Factor TIME** | **Mean POMS-bsl** | **Mean POMS-end** |
| --- | --- | --- | --- |
| T  D  A  V  S  C | F (1, 78) = 23.28; *p* < 0.0001  F (1, 78) = 41.41; *p* < 0.0001  F (1, 78) = 22.81; *p* < 0.0001  F (1, 78) = 8.842; *p* < 0.005  F (1, 78) = 53.82; *p* < 0.0001  F (1, 78) = 34.63; *p* < 0.0001 | 6.35  7.80  4.74  17.1  5.74  10.2 | 4.03  3.95  2.07  16.0  3.27  6.59 |

**Legend.** POMS-bsl: basal Profile of Mood States with, as sub-scales, T: Tension/Anxiety, D: Depression/Dejection, A: Anger/Hostility, V: Vigor/Activity, S: Fatigue/Inertia and C: Confusion/Bewilderment; POMS-end: same as above assessed at the end of the experimental session
